# TEMPO-oxidised nanocellulose hydrogels and self-standing films derived from bacterial cellulose nanopaper

**DOI:** 10.1039/d1ra04190h

**Published:** 2021-08-23

**Authors:** Kris Y. Yang, Daniela Wloch, Koon-Yang Lee

**Affiliations:** Department of Aeronautics, Imperial College London, South Kensington Campus London SW7 2AZ UK koonyang.lee@imperial.ac.uk; Institute for Molecular Science and Engineering, Imperial College London SW7 2AZ UK

## Abstract

Hydrogels derived from TEMPO-oxidised cellulose nanofibrils (TOCNs) are not robust and inherently water unstable if the TOCNs are not crosslinked or coated with a water-swellable polymer. Furthermore, the manufacturing of self-standing TOCN films is still a challenge due to the small TOCN diameter and the viscosifying effect of TOCNs. Here, we report the TEMPO-mediated oxidation of bacterial cellulose (BC) nanopaper as a route to produce robust and water stable TOCN hydrogels without the need of additional additives or crosslinking steps. Pristine BC pellicle was first press-dried into a dried and well-consolidated BC nanopaper, followed by TEMPO-oxidation at various NaClO concentrations. The oxidation reaction introduced carboxylate moieties onto the exposed BC nanofibrils within the nanopaper network structure. This then led to the expansion and swelling of the nanopaper into a hydrogel. A swelling ratio of up to 100 times the original thickness of the BC nanopaper was observed upon TEMPO-oxidation. The water retention value of the TEMPO-oxidised BC hydrogels was also found to increase with increasing carboxylate content. These TEMPO-oxidised BC hydrogels were found to be robust and water-stable, even under prolonged (>1 month) magnetic stirring in water. We further showed that high grammage self-standing TOCN films (100 g m^−2^) can be fabricated as simple as press-drying these water stable TEMPO-oxidised BC hydrogels without the need of vacuum-assisted filtration or slow-drying, which is typically the rate-limiting step in the manufacturing of TOCN films.

## Abstract 2

TEMPO-mediated oxidation of well-consolidated bacterial cellulose (BC) nanopaper derived from pristine BC pellicle as a route to produce robust and water stable nanocellulose hydrogels.

## Graphical abstract

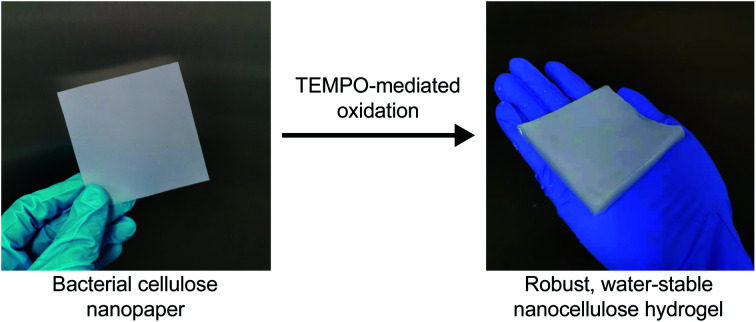

## Introduction

TEMPO [(2,2,6,6-tetramethylpiperidin-1-yl)oxyl] mediated oxidation of cellulose is a regioselective oxidation of the C6 primary hydroxyl groups of native cellulose using sodium bromide (NaBr) and sodium hypochlorite (NaClO) as the regenerating oxidants.^1^ The oxidation reaction introduces hydrophilic sodium carboxylate groups onto the surface of individual cellulose fibrils. This then gives rise to strong electrostatic repulsions between the TEMPO-oxidised cellulose fibrils in water. Consequently, simple mechanical agitation of TEMPO-oxidised cellulose in water is sufficient to individualise them, producing high aspect ratio TEMPO-oxidised cellulose nanofibrils (TOCNs) with a uniform lateral width of 3–4 nm and several micrometres in length.^2–4^ This simple and energy efficient method of producing cellulose nanofibrils has created a new wave of research in nanocellulose science and technology. Since the first report on the TEMPO-mediated oxidation of native cellulose,^2^ various research effort has been poured into the exploitation of TOCNs for packaging,^5^ wound dressing,^6,7^ energy storage,^8^ filters for separation,^9^ as well as nano-reinforcement for polymers.^1^

Engineer TOCNs into a robust and water-stable hydrogel can significantly broaden their applications particularly in drug delivery and tissue engineering. For example, a hydrogel used for wound dressing requires good mechanical properties as it also acts as a rigid protective layer.^10^ In many tissue engineering applications, the hydrogel must withstand the external load during the generation and culture of the hydrogel construct.^11,12^ Although a TOCN-in-water suspension exhibits a “hydrogel-like” character at concentration as low as 0.1%,^13^ it is not robust and water-stable. Such “hydrogel-like” TOCN-in-water suspension can be easily disrupted either through simple shear due to its shear-thinning behaviour^14,15^ or simple mechanical agitation in excess water.

Various approaches have been developed to overcome the inherent lack of robustness and water stability of the “hydrogel-like” TOCN-in-water suspension. The carboxylate groups of TOCNs can be protonated at low pH (around 2) to force the TOCNs to aggregate.^13^ Macroscopically, this leads to the formation of a TOCN hydrogel with better water stability than at neutral pH. TOCNs can be chemically crosslinked with epichlorohydrin to create a water-stable covalently bound network of TOCNs.^16^ Water-stable TOCN hydrogels can also be produced *via* the ionic crosslinking of TOCNs with divalent and trivalent cations.^17,18^ A composite approach can also be taken. TOCNs can be coated with a water swellable polymer, such as hemicellulose,^19^ poly(ethylene glycol) diacrylate^20^ or gelatin^21^ to form a water stable TOCN hydrogel. It should be noted however that this approach is at the expense of altering the surface chemistry of the TOCNs, which may be undesirable if the carboxyl functionality is required for downstream tissue engineering applications.

Microbially synthesised cellulose, more commonly known as bacterial cellulose (BC), is an ultrapure form of cellulose synthesised by cellulose-producing *Komagataeibacter* through the fermentation of low molecular weight sugars.^22^ It is produced as a pellicle consisting of a three-dimensional network of cellulose nanofibrils with lateral width and thickness of 30–50 nm and 6–10 nm, respectively.^23^ Even though BC pellicle is essentially a hydrogel that is suitable for drug delivery and support cellular growth,^24^ hydrophobic bioactive molecules do not bind to the surface of BC.^25^ In this context, the introduction of carboxyl functionality through TEMPO-mediated oxidation is useful to allow for the chemical functionalisation of BC with hydrophobic bioactive molecules, such as curcumin.^26^ Here, we report a simple method to fabricate robust and water stable TEMPO-oxidised BC hydrogels derived from dried and well-consolidated BC nanopaper. A mechanism of hydrogel formation from BC nanopaper is proposed based on experimental observations. We also demonstrate that self-standing TOCN films can be produced as simple as press-drying the TEMPO-oxidised BC hydrogels without the need of a challenging vacuum-assisted filtration or slow water evaporation step.

## Experimental section

### Materials

BC pellicle (dry grammage ∼ 100 g m^−2^) with a water content of 99 wt% was purchased from a commercial retailer (Vietcoco International Co. Ltd, Ho Chi Minh City, Vietnam). It was purified following our previously described protocol prior to subsequent use.^27^ Sodium hydroxide pellets (AnalaR NORMAPUR, purity > 99%), sodium hypochlorite solution (GPR RECTAPUR, 12% Cl_2_), hydrochloric acid (AnalaR NORMAPUR, 37%) and sodium borohydride (BioXtra, purity ≥ 99%) were purchased from VWR International Ltd (Lutterworth, UK). (2,2,6,6-Tetramethylpiperidin-1-yl)oxyl (TEMPO) (Aldrich, purity ≥ 98%), sodium bromide (BioXtra, purity ≥ 99%) and sodium chloride (Riedel-de-Haën, purity ≥ 99%) were purchased from Sigma-Aldrich (Dorset, UK). These chemicals were used as received without further purification.

### Preparation of BC nanopaper

To produce BC nanopaper, purified BC pellicle (80 mm × 80 mm × 10 mm) was first gently pressed between filter papers (Qualitative filter paper 413, VWR, Lutterworth, UK) to remove excess water from the surface of the pellicle. It was then sandwiched between fresh filter and blotting (Whatman Qualitative filter paper, Grade 3, GE Health Care, Buckinghamshire, UK) papers, followed by press-drying at 55 °C under a compaction force of 100 N for 10 min. The pressed BC pellicle was carefully removed, and the press-drying step was repeated with fresh filter and blotting papers each time until the weight of the press-dried BC remained constant. At this stage, the press-dried BC, now in its dried and well-consolidated form of a nanopaper with a nominal thickness of *ca.* 80 μm, was trimmed to dimensions of 70 mm × 70 mm prior to subsequent use.

### TEMPO-mediated oxidation of BC nanopaper

TEMPO-mediated oxidation of BC nanopaper was performed following the protocol for TEMPO-oxidising cellulose pulp described by Saito *et al.*^3^ Briefly, 49.5 mg of TEMPO and 275 mg of NaBr were first dissolved in 825 mL of deionised water under magnetic stirring. BC nanopaper (0.55 g) was then suspended in this solution. To this, the desired amount of NaClO solution, corresponding to 10, 20 and 30 mmol of NaClO per g of dry BC, respectively, was added slowly to initiate the oxidation reaction. The pH of the reaction was monitored (HI-2550, Hanna Instruments, UK) and maintained at a value of 10 through the titration of 0.5 M sodium hydroxide. The oxidation reaction was conducted for 5 h at room temperature. After the reaction, the TEMPO-oxidised BC, now in the form of a swollen hydrogel, was carefully removed from the reaction medium and rinsed with fresh deionised water until neutral pH was attained. The TEMPO-oxidised BC hydrogel was then reduced with NaBH_4_ (0.1 g of NaBH_4_ per g of dry BC) by suspending the TEMPO-oxidised BC hydrogel in 825 mL of deionised water with NaBH_4_ dissolved for 3 h at room temperature under magnetic stirring to convert any remaining aldehydes and ketones to hydroxyl groups.^28^ This reduction step was carried out to prevent heat-induced discolouration of TEMPO-oxidised BC hydrogel. After this reduction reaction, the TEMPO-oxidised BC hydrogel was rinsed with deionised water until neutral pH was attained. The reduced TEMPO-oxidised BC hydrogel was then subjected to an ion-exchange treatment to convert the sodium carboxylate groups to free carboxyl groups. The ion-exchange treatment was performed by suspending the reduced TEMPO-oxidised BC hydrogel in 825 mL of HCl solution at a pH = 1 for 1 h under magnetic stirring.^29^ After which, the ion-exchanged TEMPO-oxidised BC hydrogel was rinsed thoroughly with deionised water until a neutral pH was attained. In some experiments, the TEMPO-oxidised BC hydrogels were press-dried under a force of 9 kN at 105 °C for 30 min between filter and blotting papers in a heated hydraulic press (4122CE, Carver Inc., Wabach, IN, USA) into self-standing TEMPO-oxidised BC films.

## Materials characterisation

### Carboxylate content of TEMPO-oxidised BC hydrogels

The carboxylate content of the TEMPO-oxidised BC was determined using conductometric titration method as described by Saito and Isogai^2^ and Katz *et al.*^30^ Briefly, 0.1 g (dry basis) of TEMPO-oxidised BC was blended (Optimum 9400, Froothie Ltd, Cranleigh, UK) in 200 mL of deionized water to first produce a homogenous TEMPO-oxidised BC-in-water suspension. 10 mL of 0.01 M NaCl solution was then added to this suspension. The pH of the suspension was adjusted to 3 through the titration of 0.1 M HCl. A 40 mM NaOH solution was then pipetted at the rate of 0.2 mL min^−1^ until the pH of the TEMPO-oxidised BC suspension reached a value of 11. Throughout the NaOH titration process, the conductivity of the suspension was recorded (HI-2550, Hanna Instruments, UK) and the carboxylate content of the TEMPO-oxidised BC hydrogels was calculated based on the conductivity of the weak acid groups. All measurements were duplicated.

### Internal morphology of BC nanopaper and (TEMPO-oxidised) BC hydrogels

Scanning electron microscopy (SEM) was used to investigate the internal morphology of the samples. It was conducted using a large chamber scanning electron microscope (Hitachi S-3700N, Tokyo, Japan) operating at an accelerating voltage of 10 kV. Prior to SEM, the hydrogels were freeze-dried (Alpha 1-2 LDplus, Martin Christ, Osterode, DE). The samples were then mounted onto aluminium stubs using carbon tabs and sputter coated with Au (Agar auto sputter coater, Stansted, UK) for 20 s using a coating current of 40 mA.

### Water retention value (WRV) of TEMPO-oxidised BC hydrogels

The WRV of the TEMPO-oxidised BC hydrogels was determined using simple mass gain measurement. To determine the wet mass of the TEMPO-oxidised BC hydrogel (*m*_w_), the hydrogel was first immersed in excess deionised water for 72 h until an equilibrium was reached. Vacuum filtration was then used to drain away the excess until no dripping of water from the TEMPO-oxidised BC hydrogel was observed. The hydrogel was then sandwiched between filter papers and press-dried under a force of 9 kN at 105 °C for 30 min in a heated hydraulic press (4122CE, Carver Inc., Wabach, IN, USA) to obtain the dry mass of TEMPO-oxidised BC (*m*_d_) in the hydrogel. The WRV was calculated from
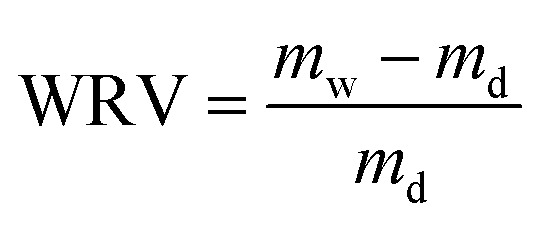


### Crystallinity of (TEMPO-oxidised) BC

The X-ray diffraction (XRD) patterns of the (TEMPO-oxidised) BC were characterised on film samples using an X-ray diffractometer (PANalytical X'pert Pro, PANalytical Ltd, Cambridge, U.K.). The measurements were taken between 2*θ* of 10° and 28° using a step size of 0.05° and a scan speed of 0.1° s^−1^. The diffractometer was equipped with a 1.54 Å Cu Kα X-ray source. The crystallinity (*χ*_c_) of the samples was calculated based on the area under the XRD curve using the following equation after baseline correction and peak deconvolution
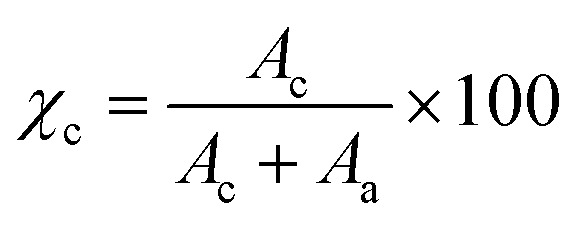
where *A*_c_ and *A*_a_ are the areas under the XRD curve ascribed to the crystalline and amorphous parts of cellulose, respectively.

### Porosity of (TEMPO-oxidised) BC films

The absolute density (*ρ*_A_) of TEMPO-oxidised BC was determined using He pycnometry (Accupyc II 1340, Micromeritics Ltd, Hexton, UK). The *ρ*_A_ of neat BC was taken as 1.60 g cm^−3^ based on data presented in the literature.^31^ To evaluate the envelope density of the (TEMPO-oxidised) BC films (*ρ*_E_), a micrometre was used to first measure the thickness of the films. With the thickness known, the envelope volume was determined and *ρ*_E_ was calculated by taking the ratio between the mass and the envelope volume of the films. The porosity (*P*) of the films was then obtained using the following equation
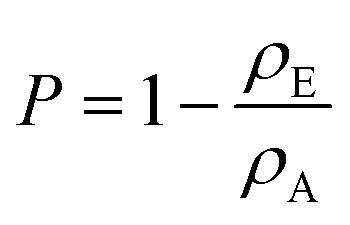


### Tensile properties of (TEMPO-oxidised) BC films

Tensile testing of the films was conducted in accordance with ASTM D632-14. Rectangular shaped test specimens were cut from the (TEMPO-oxidised) BC films using a manual cutting press (ZCP 020, Zwick Roell Ltd, Herefordshire, UK). The test specimens possessed an overall length of 40 mm and a width of 5 mm. Tensile test was conducted using a micro-tensile tester (model MT-200, Deben UK Ltd, Woolpit, UK) equipped with a 200 N load cell. After mounting the test specimen on the micro tensile tester, the exposed specimen length was 25 mm. Prior to loading the specimen under uniaxial tension, a speckle pattern was marked on the surface of the test specimen and the strain of the test specimen was evaluated by monitoring the movement of this pattern using a noncontact video extensometer (iMetrum Ltd, Bristol, UK). All test specimens were loaded with a crosshead displacement speed of 0.5 mm min^−1^, which corresponded to a strain rate of 3 × 10^−4^% s^−1^. A total of 5 specimens were tested for each sample.

## Results and discussion

**Fig. 1:**
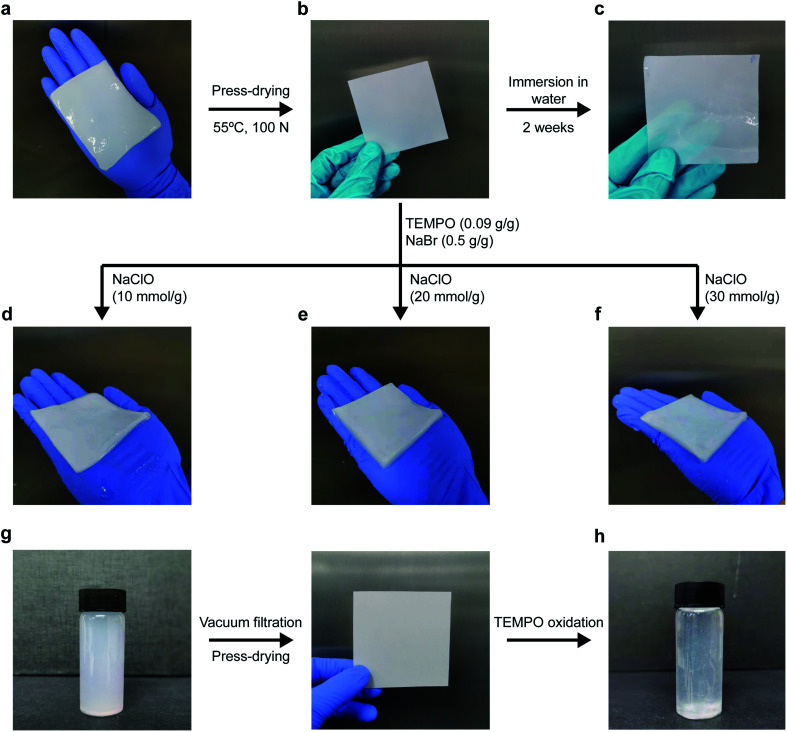
(a) BC pellicle containing *ca.* 99 wt% water and the press-drying of this BC pellicle produces (b) a dried and well-consolidated BC nanopaper. (c) BC nanopaper after immersion in water at room temperature for two weeks, showing that it cannot be rehydrated into its original pellicle form. The visual appearance of TEMPO-oxidised BC hydrogels produced from the oxidation of BC nanopaper using (d) 10 mmol, (e) 20 mmol and (f) 30 mmol of NaClO per g of dry BC, respectively. (g) A homogenous suspension of BC-in-water, followed by vacuum filtration and press-drying to produce BC nanopaper. (h) The outcome of TEMPO-mediated oxidation of BC nanopaper produced from disintegrated BC pellicle using an NaClO concentration of 10 mmol per g of dry BC.

The press-drying of BC pellicle (Fig. 1a) produces a dried and well-consolidated network of BC nanofibrils, more commonly known as BC nanopaper (Fig. 1b). When BC nanopaper was immersed in water, it did not rehydrate into its original pellicle form (Fig. 1c). This can be attributed to the highly crystalline nature of BC^32,33^ that prevented intra-fibril swelling and inter-fibril hornification, *i.e.*, the irreversible bond formation between adjacent cellulose fibrils upon drying.^34,35^ For the latter, the high surface area of BC nanofibrils (measured to be 41 m^2^ g^−1^)^36^ leads to a large number of contact or physical crosslinking points between adjacent BC nanofibrils *via* hydrogen bonding. Thus, the BC nanopaper remained stable in water. When BC nanopaper was subjected to TEMPO-mediated oxidation, the nanopaper swelled into a hydrogel (Fig. 1d–f, corresponding to TEMPO-mediated oxidation of BC nanopaper using 10, 20 and 30 mmol of NaClO per g of dry BC, respectively). These TEMPO-oxidised BC hydrogels were found to be robust and water stable as they did not disintegrate under prolonged (>1 month) magnetic stirring in water.

To ascertain whether TEMPO-mediated oxidation is necessary to cause the swelling of the BC nanopaper into a hydrogel, BC nanopaper was immersed in NaClO/NaBr, TEMPO/NaBr and NaClO/TEMPO solutions under magnetic stirring for 5 h. No swelling of the BC nanopaper was observed in these solutions, confirming that TEMPO-mediated oxidation is indeed required to swell the BC nanopaper into a hydrogel. In addition to the direct press-drying of a BC pellicle, nanopaper can also be produced by first disintegrating BC pellicle using a blender to first produce a homogenous suspension of BC-in-water, followed by filtration and press-drying^31,37–39^ (Fig. 1g). Therefore, we further investigated whether the same swelling effect could also be achieved by the TEMPO-mediated oxidation of BC nanopaper produced *via* this method. To our surprise, the BC nanopaper disintegrated during the oxidation reaction instead of swelling into a water stable hydrogel (Fig. 1h), even at the lowest NaClO concentration used in this work. These results suggest the importance of the starting BC network.

**Fig. 2:**
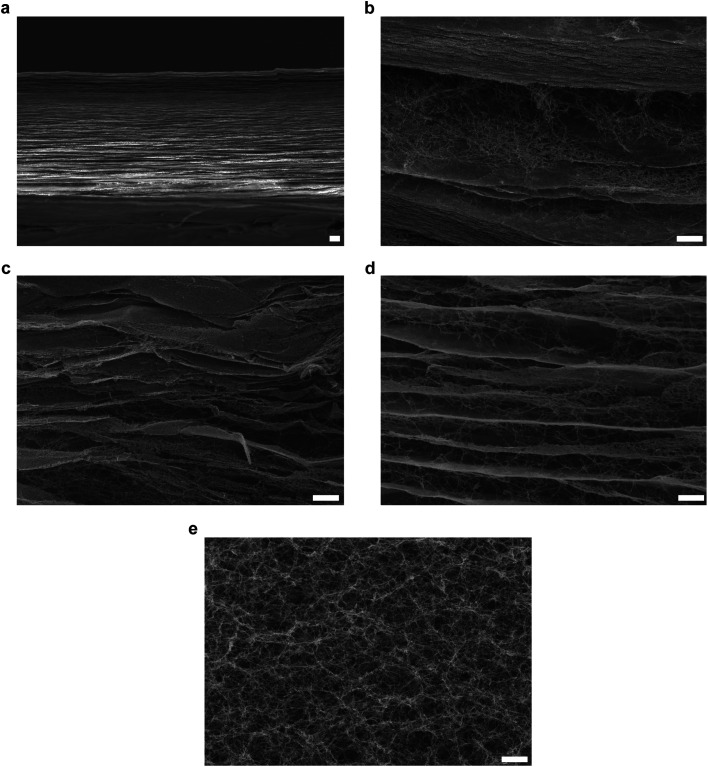
SEM micrographs of (a) neat BC nanopaper, and TEMPO-oxidised BC hydrogels modified with (b) 10 mmol, (c) 20 mmol and (d) 30 mmol of NaClO per g of dry BC, as well as (e) neat BC pellicle. Scale bar = 4 μm.

To further investigate the mechanism behind the swelling of the BC nanopaper made from pristine BC pellicle into a hydrogel upon TEMPO-mediated oxidation, SEM was conducted. Morphologically, BC nanopaper possesses a layered structure consisting of multiple thin nanocellulose sheets (Fig. 2a). Similar layered structure has also been observed by numerous researchers.^40–42^ The internal morphology of the TEMPO-oxidised BC hydrogels, on the other hand, contains both the thin nanocellulose sheets as well as loose BC nanofibrils in between the sheets (Fig. 2b–d). It is well-known that at the contact or physical crosslinking points between adjacent BC nanofibrils, the hydroxyl groups remained inaccessible.^43–45^ Therefore, the oxidation reaction of the BC nanopaper made from pristine BC pellicle can only proceed from the accessible hydroxyl groups of the exposed nanofibrils within the nanopaper network structure, which are found in between the thin nanocellulose sheets. As the reaction progresses, more water molecules penetrate the nanopaper structure due to the hydrophilicity of sodium carboxylate groups. This then gives rise to electrostatic repulsions between the TEMPO-oxidised BC nanofibrils, causing the layered structure of the BC nanopaper to expand and swell into a hydrogel. Pristine BC pellicle consists of a pseudo continuous network of BC nanofibrils (Fig. 2e), hypothesised to be a result of continuous fibril synthesis during cell division.^46–48^ As a result, the TEMPO-oxidation of BC nanopaper made from pristine BC pellicle produces a water stable TEMPO-oxidised BC hydrogel. Disintegrating the BC pellicle disrupted this continuity.^31^ Consequently, the BC nanopaper made from disintegrated BC pellicle disintegrated upon TEMPO-oxidation.

**Fig. 3:**
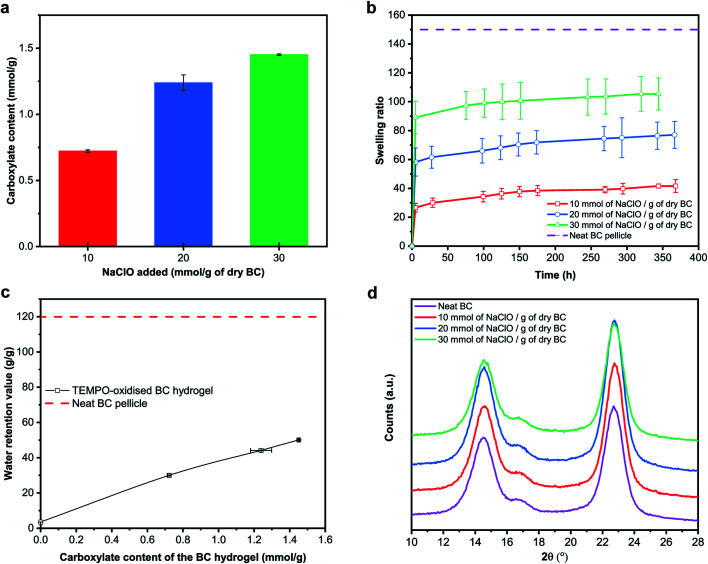
(a) Carboxylate content, (b) swelling ratio and (c) water retention value of the TEMPO-oxidised BC hydrogels, as well as (d) X-ray diffraction patterns of the (TEMPO-oxidised) BC.

Fig. 3a summarises the carboxylate content of TEMPO-oxidised BC hydrogels as a function of NaClO concentration added. A carboxylate content of 0.72 mmol g^−1^ was achieved for BC nanopaper TEMPO-oxidised with an NaClO concentration of 10 mmol g^−1^. Increasing the concentration of NaClO to 20 mmol g^−1^ and 30 mmol g^−1^ increased the carboxylate content to 1.24 mmol g^−1^ and 1.45 mmol g^−1^, respectively. Wu *et al.*^49^ studied the efficiency of TEMPO-mediated oxidation of freeze-dried BC at an NaClO concentration of 10 mmol g^−1^ as a function of reaction time. At a reaction time similar to ours, the authors obtained a carboxylate content of 1.6 mmol g^−1^; double that of what we have obtained at the same NaClO concentration. As a reference, we also performed TEMPO-oxidation directly on never-dried BC pellicle at an NaClO concentration of 10 mmol g^−1^. A carboxylate content of 1.32 mmol g^−1^ was obtained. However, this TEMPO-oxidised BC hydrogel was not robust and disintegrated easily in water.

Nevertheless, these results corroborated with the reduced hydroxyl groups accessibility of BC nanopaper due to interfibril hornification and the proposed mechanism that the oxidation reaction can only proceed from the exposed BC nanofibrils in the nanopaper structure. The efficiency of TEMPO-oxidation of cellulose is a function of pH, reaction time and NaClO-to-cellulose concentration used but the influence of the accessible hydroxyl groups on the resulting carboxylate content has rarely been commented on. Saito *et al.*^3^ previously reported that both never-dried and once-dried cellulose pulp are equally susceptible to TEMPO-oxidation reaction. However, this did not seem to be the case for nanocellulose, presumably due to the strong interfibril hornification between the nanofibrils compared to cellulose pulp.

Fig. 3b and c show the swelling ratio of the BC nanopaper due to TEMPO-mediated oxidation and the water retention value (WRV) of the TEMPO-oxidised BC hydrogels with different carboxylate contents, respectively. It can be seen from these figures that both the swelling ratio and WRV increase with increasing carboxylate content. WRV reflects the swelling of the BC nanopaper into the water stable TEMPO-oxidised BC hydrogels and is influenced by charge density. It should be noted however that both the swelling ratio and WRV did not recover to the values of the original BC pellicle. The X-ray diffraction patterns of the (TEMPO-oxidised) BC are shown in Fig. 2d. The peaks at 14.5°, 16.5° and 22.5° correspond to the diffraction planes of (100), (010) and (110) for cellulose-Iα, respectively.^50^ No peak shift or appearance of new peaks was observed. The degree of crystallinity of the (TEMPO-oxidised) BC was calculated to be ∼88% for all samples. These results implied that the TEMPO-oxidation of BC occurred only on the surface of the BC nanofibrils, consistent with the findings by Saito *et al.*^2,51^

**Fig. 4:**
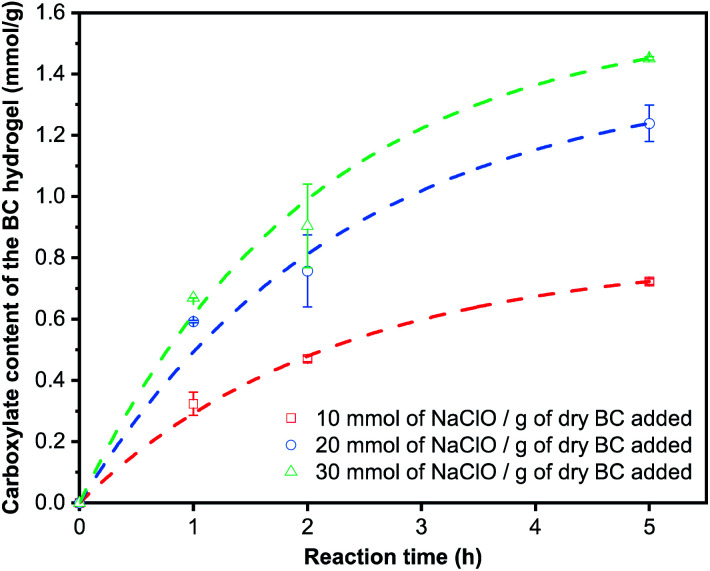
Carboxylate content of the TEMPO-oxidised BC hydrogels as a function of reaction time.

We further ascertain whether the TEMPO-mediated oxidation of BC nanopaper is kinetically or diffusion limited at the start of the oxidation reaction. This is because BC nanopaper possesses a low water permeance.^52^ The oxidation reaction may first proceed from the surface of the BC nanopaper. This then causes initial swelling only on the surface of the BC nanopaper, exposing the hydroxyl groups of the nanocellulose sheets immediately below the surface for oxidation until the whole layered structure of the nanopaper expands and swells into a TEMPO-oxidised BC hydrogel. If the TEMPO-mediated oxidation of BC nanopaper is diffusion limited at the start of the oxidation reaction, the initial rate of reaction will not be a function of NaClO concentrations added. On the other hand, if the TEMPO-mediated oxidation of BC nanopaper is kinetically limited at the start, the initial rate of reaction will be influenced by the concentration of NaClO added. Therefore, the TEMPO oxidation reaction was conducted as a function of reaction time at various NaClO concentrations (Fig. 4). It was found that the initial carboxylate content of the TEMPO-oxidised BC was a function of NaClO concentration used. This implies that the initial rate of reaction is kinetically limited and the oxidation reaction proceeds uniformly within the BC nanopaper structure instead of starting from the surface of the BC nanopaper and proceed slowly into the bulk.

**Fig. 5:**
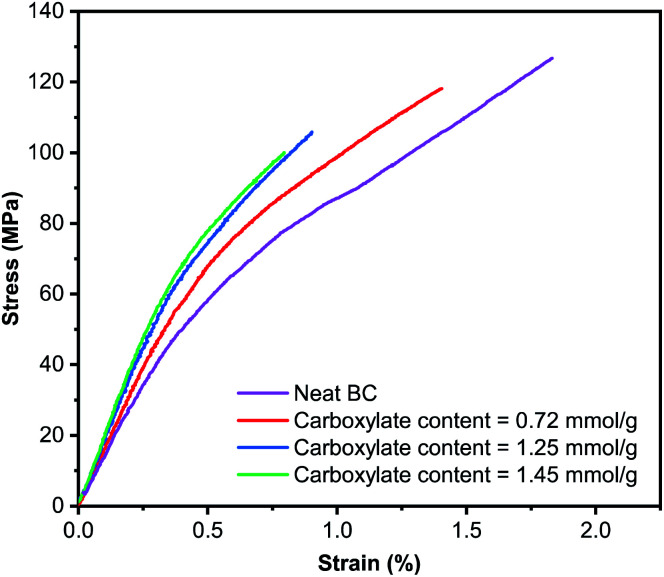
Representative tensile stress–strain curves of the various (TEMPO-oxidised) BC films.

The robust and water-stable TEMPO-oxidised BC hydrogels were then press-dried into self-standing TEMPO-oxidised BC films. In general, the production of self-standing TOCN film starts with a suspension of TOCN-in-water. This is then followed by vacuum filtration.^53–55^ Due to the small nanofibril diameter, homogeneity of the TOCN suspension and the viscosifying effect of TOCNs, filtering a suspension of TOCNs remains a challenge. Multivalent salts could be added to reduce the Debye length of TOCNs, forcing their aggregation in water to reduce filtration time.^56^ Water evaporation is also a commonly used technique to fabricate self-standing TOCN films.^29,57,58^ However, long drying times are required as low drying temperature is necessary to produce high quality uniform TOCN films. Therefore, the grammage of TOCN films produced is typically low. Here, the possibility of first producing a robust and water stable TOCN hydrogel allowed for the production of self-standing TOCN film as simple as press drying without the need of vacuum assisted filtration or water evaporation. The porosity of the TEMPO-oxidised BC films was found to be ∼20% (Table 1). By contrast, neat BC film possesses a higher porosity of ∼35%. Fig. 5 presents the representative stress–strain curves of the (TEMPO-oxidised) BC films tested under uniaxial tension. After the initial elastic response, all samples yielded prior to catastrophic failure, characterised by a sudden load drop to zero once peak stress was reached.

**Table 1:** Tensile properties of the various (TEMPO-oxidised) BC films. *ρ*_A_, *P*, *E*, *σ*, *ε* and *U*_T_ denote the absolute density, porosity, Young's modulus, tensile strength, strain-at-failure and work of fracture of the films, respectively

Film	Carboxylate content [mmol g^−1^]	*ρ*_A_ [g cm^−3^]	*P* [%]	*E* [GPa]	*σ* [MPa]	*ε* [%]	*U*_T_ [MJ m^−3^]
Neat BC	—	1.60 ± 0.01^a^	35.3 ± 3.1	15.4 ± 0.8	122 ± 9	1.60 ± 0.37	1.15 ± 0.32
TEMPO-oxidised BC	0.72 ± 0.01	1.47 ± 0.01	23.5 ± 0.8	15.9 ± 1.0	119 ± 10	1.48 ± 0.22	1.05 ± 0.26
	1.24 ± 0.06	1.50 ± 0.01	20.7 ± 0.4	18.5 ± 0.9	102 ± 12	0.89 ± 0.26	0.57 ± 0.23
	1.45 ± 0.01	1.50 ± 0.01	19.5 ± 0.8	18.9 ± 0.9	91 ± 9	0.79 ± 0.11	0.40 ± 0.12

a Data obtained from Santmarti *et al.*^31^

Neat BC film possesses a tensile modulus of 15.4 GPa (Table 1). By increasing the carboxylate content of the resulting TEMPO-oxidised BC films, the tensile modulus increases from 15.9 GPa for TEMPO-oxidised BC film with a carboxylate content of 0.72 mmol g^−1^ to nearly 19 GPa for TEMPO-oxidised BC film with a carboxylate content of 1.45 mmol g^−1^. However, this increase is at the expense of the tensile strength of the TEMPO-oxidised BC films. The tensile strength of TEMPO-oxidised BC films decreased from 119 MPa to only 91 MPa when the carboxylate content increased from 0.72 mmol g^−1^ to 1.45 mmol g^−1^. Neat BC film, on the other hand, possesses a tensile strength of 122 MPa. The strain-at-failure of neat BC film was found to be 1.60% (Table 1). TEMPO-oxidised BC films were found to be brittle. With increasing carboxylate content, the strain-at-failure of the TEMPO-oxidised BC film decreased progressively to only 0.79%. Correspondingly, the work-of-fracture of the (TEMPO-oxidised) BC films decreased with increasing carboxylate content. These results can be attributed to the difference in interfibrillar bond strength between the (TEMPO-oxidised) BC nanofibrils. For TEMPO-oxidised BC nanofibrils, two carboxyl moieties form two hydrogen bonds with each other. However, two hydroxyl groups in neat BC nanofibrils can only form one hydrogen bond with each other. As a result, the tensile modulus of TEMPO-oxidised BC films is higher than that of neat BC films and increases with increasing carboxylate content. This also reduces the plasticity of the TEMPO-oxidised BC films, increasing their brittleness and reducing their tensile strength.

## Conclusions

In summary, we demonstrated a simple method to fabricate robust and water stable TOCN hydrogels based on the TEMPO-mediated oxidation of dried and well-consolidated BC nanopaper. We found that this observation is unique only to nanopaper press-dried from pristine BC pellicle. The direct TEMPO-mediated oxidation of BC nanopaper produced from disintegrated BC pellicle, however, leads to the destruction of the nanopaper, forming a homogenous TEMPO-oxidised BC nanofibril-in-water suspension. The initial rate of TEMPO-mediated oxidation of BC nanopaper was observed to be kinetically limited. The tensile properties of TEMPO-oxidised BC films produced from the press drying of TEMPO-oxidised BC hydrogels were found to be a strong function of carboxylate content. Increasing the carboxylate content of TEMPO-oxidised BC film leads to an increase in tensile modulus (from 15 GPa to nearly 19 GPa) but at the expense of tensile strength (from 122 MPa to only 91 MPa) and strain-at-failure (1.60% to 0.79%). This is a result of increased strength of hydrogen bonding within the TEMPO-oxidised BC films due to the presence of carboxyl functionality.

## Conflicts of interest

There are no conflicts to declare.

## Supplementary Material
